# A Harmonized International Database of More Than 10,000 Pediatric Renal Tumor Patients From 30 Years of SIOP-RTSG Studies

**DOI:** 10.1200/CCI-25-00390

**Published:** 2026-06-23

**Authors:** Prakriti Roy, Ingrid Schut, Norbert Graf, Arnauld Verschuur, Tanzina Chowdhury, Filippo Spreafico, Jesper Brok, Gema L. Ramirez-Villar, Beatriz de Camargo, Jan Godzinski, Gordan Vujanic, Patrick Melchior, Ria Koolma, Reem Al-Saadi, Manfred Gessler, Catriona Duncan, Jens-Peter Schenk, Rhoikos Furtwängler, Helene S. Bonnange, Severine Metzger, Danny Baars, Kathy Pritchard-Jones, Marry M. van den Heuvel-Eibrink, Harm van Tinteren

**Affiliations:** ^1^ Princess Maxima Center for Paediatric Oncology, Utrecht, the Netherlands; ^2^ Department of Paediatric Oncology and Hematology, Saarland University, Homburg, Germany; ^3^ Department of Paediatric Oncology, Hôpital d'Enfants de la Timone, Marseille, France; ^4^ Department of Haematology and Oncology, Great Ormond Street Hospital for Children NHS Foundation Trust, London, UK; ^5^ Department of Medical Oncology and Hematology, Paediatric Oncology Unit, Fondazione IRCCS Instituto Nazionale dei Tumori, Milan, Italy; ^6^ Department of Paediatric Oncology and Hematology, Rigshospitalet, Copenhagen, Denmark; ^7^ Department of Paediatric Oncology, Hospital Universitario Virgen del Rocío, Seville, Spain; ^8^ Research Center, Instituto Nacional do Cancer, Rio de Janeiro, Brazil; ^9^ Department of Paediatric Surgery, Marciniak Hospital, Wroclaw, Poland; ^10^ Department of Paediatric Traumatology and Emergency Medicine, Wroclaw Medical University, Wroclaw, Poland; ^11^ Department of Pathology, Sidra Medicine and Weill Cornell Medicine, Doha, Qatar; ^12^ Department of Radiation Oncology, Saarland University Hospital, Homburg, Germany; ^13^ Developmental Biology and Cancer Research and Teaching Department, University College London Great Ormond Street Institute of Child Health, London, UK; ^14^ Theodor-Boveri-Institute/Biocenter and Comprehensive Cancer Center Mainfranken, University of Würzburg, Würzburg, Germany; ^15^ Paediatric Radiology Section, Department for Diagnostic and Interventional Radiology, University Hospital Heidelberg, Heidelberg, Germany; ^16^ Paediatric Haematology and Oncology, Department of Paediatrics—Inselspital, University Bern, Bern, Switzerland; ^17^ Children and AYA Unit, Oscar Lambret Center, Lille, France; ^18^ Department of Clinical Research and Innovation, Centre Léon Bérard, Lyon, France; ^19^ Division of Child Health Utrecht, Wilhelmina Kinderziekenhuis & University of Utrecht, Utrecht, the Netherlands

## Abstract

**BACKGROUND AND SCOPE:**

Historical data sources for rare cancers are valuable for data-driven research. The International Society of Pediatric Oncology Renal Tumors Study Group (SIOP-RTSG) has been conducting clinical research on pediatric renal tumors for more than 50 years. Since 1993, data have been collected across multiple countries, using standardized paper-based case report forms that were digitally transcribed into electronic databases. Until 2019, prospectively collected data are present in nine different electronic sources from three consecutive clinical studies. More than 10,000 patient cases of kidney cancer in children are registered in totality. However, the necessary merging of numerous sources to answer a research question is repetitive, time consuming, and unsustainable in the long term.

**SOLUTION:**

By mapping and formatting the structure of these nine databases, a single database was created that was similar in design to the prospective study database. This allows the data from the past and current studies to be accessed and queried efficiently with standardized syntax and reports.

**EVALUATION:**

We validated this new database by reviewing whether uploads were complete and successful, mapped items were coded correctly, or discrepancy appeared in the export. Comparisons with retrospective reports were made to ensure the datasets were reproducible.

**RELEVANCE:**

Researchers have access to a large number of patients through this validated aggregated database. Data maintenance and extraction is efficient, and it offers the possibility to complete missing data directly into the central database. This ultimately ensures long-term availability in a secure, flexible, and responsible manner, in accordance with FAIR principles.

**HOW TO ACCESS/USE:**

Researchers can send questions to SIOP-RTSG and, if necessary, gain access to the database after short training. Access granted would depend on user's role.

## INTRODUCTION

In 2022, the International Society of Pediatric Oncology Renal Tumors Study Group (SIOP-RTSG) celebrated its 50th anniversary of international collaboration that resulted in research and guidelines to improve the survival and quality of life for children with renal tumors.^1^ During these years, seven consecutive trials and studies (including randomized controlled trials) were initiated and completed. The SIOP-RTSG has grown over the years from a small European collaboration to a global association at present with the participation from more than 33 countries/regions in Europe, South America, and Asia. Since 1993, the group has made efforts to standardize data collection, centralized management and statistical analysis of datasets from different collaborating countries.

In SIOP-93-01, study data were collected on paper-based case record forms (CRFs) and sent to be entered centrally into one of two independent databases (in Microsoft Access) located in Germany or The Netherlands. For SIOP-2001, a centrally developed database (also in Microsoft Access) was distributed as a copy, allowing independent data entry by participating countries or cohorts. In the UK-IMPORT study, local sites from the United Kingdom and Ireland sent completed paper-based CRFs to be manually entered in a central database in London. After the randomized study of SIOP 2001 was closed in 2009, some national groups adapted the existing centrally created database to their national needs or preferred self-developed systems to continue patient registration and data collection. However, the items to be collected were always coordinated internationally. Moreover, the basic CRF format and items were standard across all consecutive studies with some variation in details of the captured variables as per protocol. Once or twice a year, the data were then sent to a central location and compiled into one large dataset to perform overall data analysis. This increasingly became a repetitive, time consuming, and laborious process. In 2019, the UMBRELLA SIOP-RTSG-2016 study (UMBRELLA study) began accruing new patients.^2^ The study used for the first time a cloud-based Electronic Database Capturing (EDC) system within the SIOP-RTSG network, developed in ALEA (ALEA Clinical Services BV, Abcoude, NL). This database was used to capture data from all participating countries and sites independently, except Germany and Austria (which introduced another EDC system called ObTiMA).

**FIG 1.**
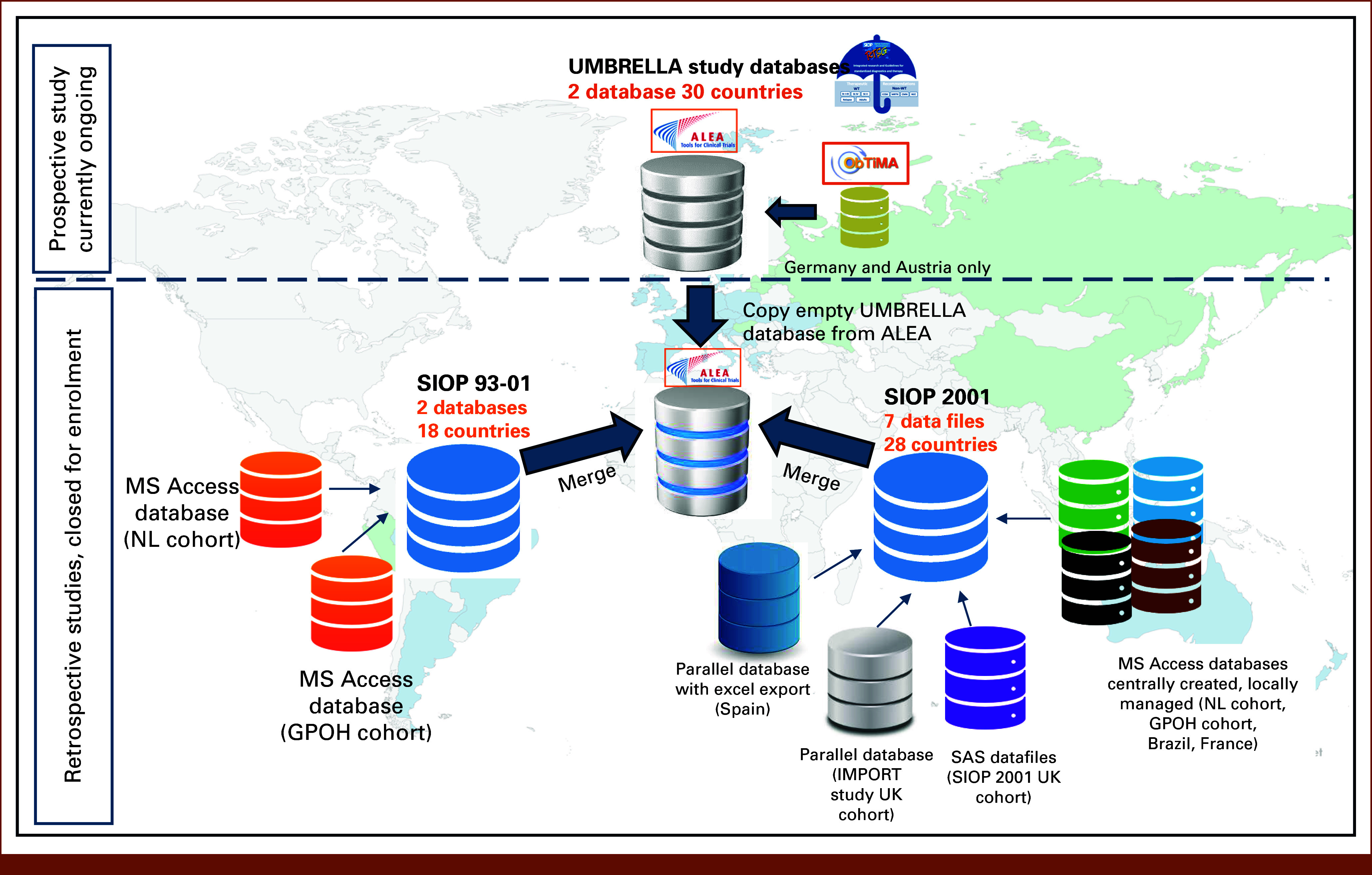
Representation of all the different data sources (n = 9) used to merge into a single database. GPOH, German pediatric oncology and hematology group, represented by Austria, Germany and Switzerland; MS Access, Microsoft Access‑based database; NL cohort, The Netherlands and additional countries/regions participating in SIOP 93-01 and SIOP 2001 studies; SAS data files, SAS analytics software compatible data files.

From a sustainability and efficiency perspective, it seemed ideal to transfer the historical data into a similar data model in a cloud-based application (Fig 1). Since most of the items in the earlier CRFs were standardized by multidisciplinary panels of SIOP-RTSG and only improvised over the years in the UMBRELLA eCRFs, it was more appropriate to transfer the historical data to a copy of the UMBRELLA study. In this article, we present the process of merging the data from SIOP-93-01, SIOP-2001, and the UK-IMPORT studies into a cloud-based EDC system and the governance plan that was developed to manage the usability of the data.

## METHODS

### International Data Collection

The core set of items to be collected was developed and improved since the 1980s by representatives of the various disciplines involved in the treatment of pediatric renal tumors from across Europe. After obtaining informed consent according to the country-specific requirements, data were collected for demographics; disease characteristics; and preoperative and postoperative chemotherapy, surgery, radiotherapy, and follow-up details, including recurrence or progression, if any, and outcome. With each new study protocol, the number of items increased as research questions changed, and new variables were added as applicable. In addition to an increasing number of items, the option was also introduced to enter multiple forms (eg, for bilateral tumors and relapses) or entering several lines in tables within a form (eg, different modalities for imaging tumor volume). This resulted in capturing a core dataset of approximately 450 variables across 20 CRFs over the consecutive protocols.

### Process of Merging the CRFs

Mapping involves aligning and harmonizing the variables of retrospective studies with the current UMBRELLA study variables and associated answer options. For mapping, all groups/cohorts were asked to submit their historical datasets by November 2023. Advice was sought from SIOP-RTSG disciplinary panels regarding some variables that were difficult to map directly, either because some items were dropped over time or because they were less or more detailed than in the current database. Principally, the UMBRELLA variables were used as default and no new questions were added. For some essential questions, the option groups were extended or recoded or reassigned to include past answer options. These minor adjustments or deviations are filed in a separate data specification document. The variables and options used for the merged historical database are available in a data file named “codebook” available in the cloud-based application.

Transformation is the process of converting the available data structure of the historical datasets to the UMBRELLA study database, which follows a data format compliant with the Clinical Data Interchange Standards Consortium Operational Data Model (CDISC ODM-XML). In SIOP-93-01, SIOP-2001, and UK-IMPORT databases, this structure was not/partially used. This involved converting multiple forms or events from a wide data format linked by a single key variable to relational data tables that are linked by one or more key variables (primary and alternate keys). In the past, for example, a change in tumor volume was captured longitudinally, at preoperative and postoperative chemotherapy time points by a single primary key, that is, the subject number. In the new format, this is done specifically for the time point, with subject number as the key variable and time of the event (before or after chemotherapy) and imaging mode being the alternate key variables. The transformations were performed in SAS (v9.4; Copyright (c) 2016 by SAS Institute Inc, Cary, NC).

Importing is the process of uploading the transformed data files to the copy of the UMBRELLA database in the cloud-based application. After the files were converted into the desired format, a new subject key was assigned to each record. The subject key was similar to the current subject identification rules in the UMBRELLA study, based on country, groups/cohorts, and previous patient number, which ensured that historical patients could be traced back. However, previously used identifying information (such as date of birth and initials) was not imported and instead stored on a separate server independent from the database. Once the mapping and transformation were complete, batch importing was performed by a unit of CRFs. Finally, groups/cohorts were asked to resubmit the most updated datasets to be imported by July 2024.

### System Validation

Validation involved checking that uploaded data were complete as in the source files and that mapped items were imported correctly. After each upload, the import log was checked, and errors were resolved to ensure all uploads were successful. For each CRF, the content was checked to identify unlikely values. If discrepancies were noted, they were queried, resolved, or clarified and a revised dataset was imported. Besides this, statisticians compared the analysis of current data against retrospective peer-reviewed publications to ensure that the datasets were reproducible. If discrepancies were not resolvable, the source data were consulted to ensure the data present are accurately captured in the merged database.

## RESULTS

The SIOP-93-01, SIOP 2001, and UK-IMPORT studies included essentially all subtypes of renal tumors. All included patients were registered after obtaining informed consent for one of the studies, and data collection adhered to the relevant ethical guidelines applicable during the study periods. All patient's personal data were pseudonymized as per the current General Data Protection Regulations (GDPR)^3^ and used in the merged dataset. Consent was obtained for registration and random assignment separately.

**FIG 2.**
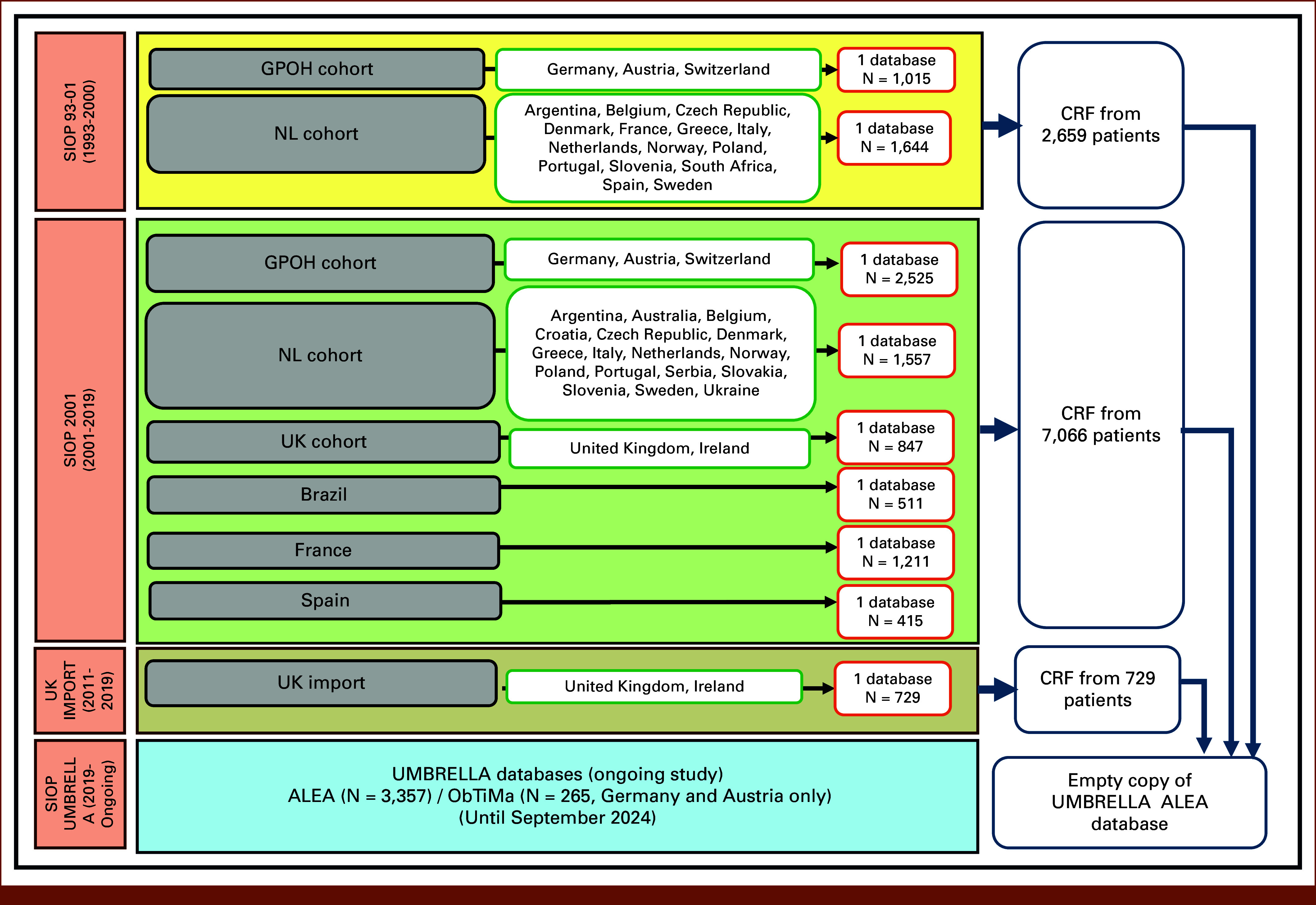
Participating countries and merged data flow. GPOH, German pediatric oncology and hematology group, represented by Austria, Germany and Switzerland; NL cohort, The Netherlands and other countries registering through the NL data office. UK cohort, cohort comprising the whole of the United Kingdom and Ireland.

The studies were designed mainly to answer questions for patients with Wilms tumor (WT) that met certain eligibility criteria, and particular subgroups were randomly assigned to different treatments. Patients eligible for random assignment were considered “trial patients” or “randomly assigned patients”. All other subgroups of patients with WT for whom approved treatment guidelines were available but were not included in the random assignment question were recorded as “protocol patients”. Patients with other renal tumors (so-called non-WTs) or those who did not fit the eligibility criteria for inclusion in the studies or protocol (eg, because of age, immediate surgery, nephroblastomatosis, extrarenal tumors, and bilateral disease with metastases) were registered as observational “study patients” or “nonprotocol” patients. Other details of the protocols are present in the linked resource^4,5^ and summarized in Appendix Table A1. The randomization for the SIOP-2001 trial was closed in December 2009. While all countries were requested to continue registering patients in the SIOP 2001 database, it was not mandated and differed per country. In the United Kingdom and Ireland, the SIOP 2001 study was closed in 2011 and the UK-IMPORT study opened in September 2012. The UK-IMPORT study used the same treatment recommendations as the SIOP-2001 study. The ongoing UMBRELLA study was opened in June 2019.^2^ SIOP-93-01 was open from 1993 to 2000 and registered 2659 patients from 18 countries. Data were collected in 2 separate databases located in local servers in Germany and The Netherlands. SIOP-2001 study was open from 2001 to 2019, and the UK-IMPORT study was open from 2012 to 2019. Together, these two studies registered 7,795 patients from 28 countries. The data were recorded in seven different databases, which were then extracted as data files in various formats (Fig 2).

This project was conceptualized in June 2021. It took 2 years to map the datasets from nine source files. These were then uploaded in 2023, and eventually updated data files were received by July 2024. In September 2024, the newly created database was locked and ready to be used for validation and data analysis. In total, currently, data from 10,454 patients from 28 countries over 26 years are present in the cloud-based application. The patient characteristics from the three studies are presented in Table 1.

**TABLE 1.** General Characteristics of Patients Registered in SIOP-RTSG Databases (Locked Dataset From September 2024)

Characteristic	N	Overall, N = 10,454^1^ (%)	RTSG Study (%)		
			SIOP9301, N = 2,659^1^	SIOP2001, N = 7,066^1^	IMPORT (UK), N = 729^1^
Protocol patients^a^	10,454				
Unilateral localized WT		6,710 (64.2)	1,685 (63.4)	4,559 (64.5)	466 (63.9)
Unilateral metastatic WT		1,164 (11.1)	210 (7.9)	840 (11.9)	114 (15.6)
Bilateral localized WT		401 (3.8)	79 (3.0)	281 (4.0)	41 (5.6)
Nonprotocol patients^b^		2,179 (20.8)	685 (25.7)	1,386 (19.6)	108 (14.8)
Randomly assigned patients^c^	947				
SIOP-2001					
AVD		276 (29)		276 (51)	0 (NA)
AV2		266 (28)		266 (49)	0 (NA)
SIOP-9301					
Two more cycles VA		210 (22)	210 (52)		
No further treatment		195 (21)	195 (48)		
Sex	10,421				
Male		4,913 (47)	1,235 (46)	3,339 (47)	339 (47)
Female		5,508 (53)	1,423 (54)	3,695 (53)	390 (53)
Missing		33	1	32	0
Patient age	10,439				
Nonmissing		10,439	2,655	7,055	729
Median (in months) (Q1, Q3)		36 (16, 59)	37 (16, 60)	35 (16, 59)	39 (18, 64)
Missing		15	4	11	0
Site primary tumor	10,422				
Right		4,686 (45)	1,200 (45)	3,157 (45)	329 (45)
Left		4,830 (46)	1,228 (46)	3,267 (46)	335 (46)
Bilateral		863 (8.3)	203 (7.7)	596 (8.5)	64 (8.8)
Extrarenal		43 (0.4)	19 (0.7)	23 (0.3)	1 (0.1)
Missing		32	9	23	0

NOTE. Median (IQR) or frequency (%).

Abbreviations: AV or VA, actinomycin D and vincristine; AVD, actinomycin D, vincristine, and doxorubicine.

a Protocol patients = Patients eligible for treatment according to protocol guidelines and random assignment.

b Nonprotocol patients (study patients) = Patients for whom no protocol guidelines were available. By definition these were patients younger than 6 months of age or needed surgical emergency or non-WT or metastatic bilateral tumor or having extrarenal tumors.

c Randomly assigned patients: Patients included for randomly assigned study questions.

Validation of the database was performed after the data were frozen for analysis in September 2024. Discrepancies on codebooks were resolved immediately. Data discrepancies, recovered missing data, and additional follow-up data are periodically resolved into the database.

The merged database will preserve the data in a secured cloud-based database and will allow easy analyses of data from past SIOP-RTSG studies. There is also the possibility to complete missing data, correct erroneous data, and add follow-up data remotely wherever ethically permissible. The database will be access controlled, which means that only trained and authorized individuals will have access to the database, and any reports from this database will be authorized by the SIOP-RTSG statistics and data management committee before delivery. The data handling access is coordinated by the SIOP-RTSG office, in close collaboration with the statistics and data management committee.

**FIG 3.**
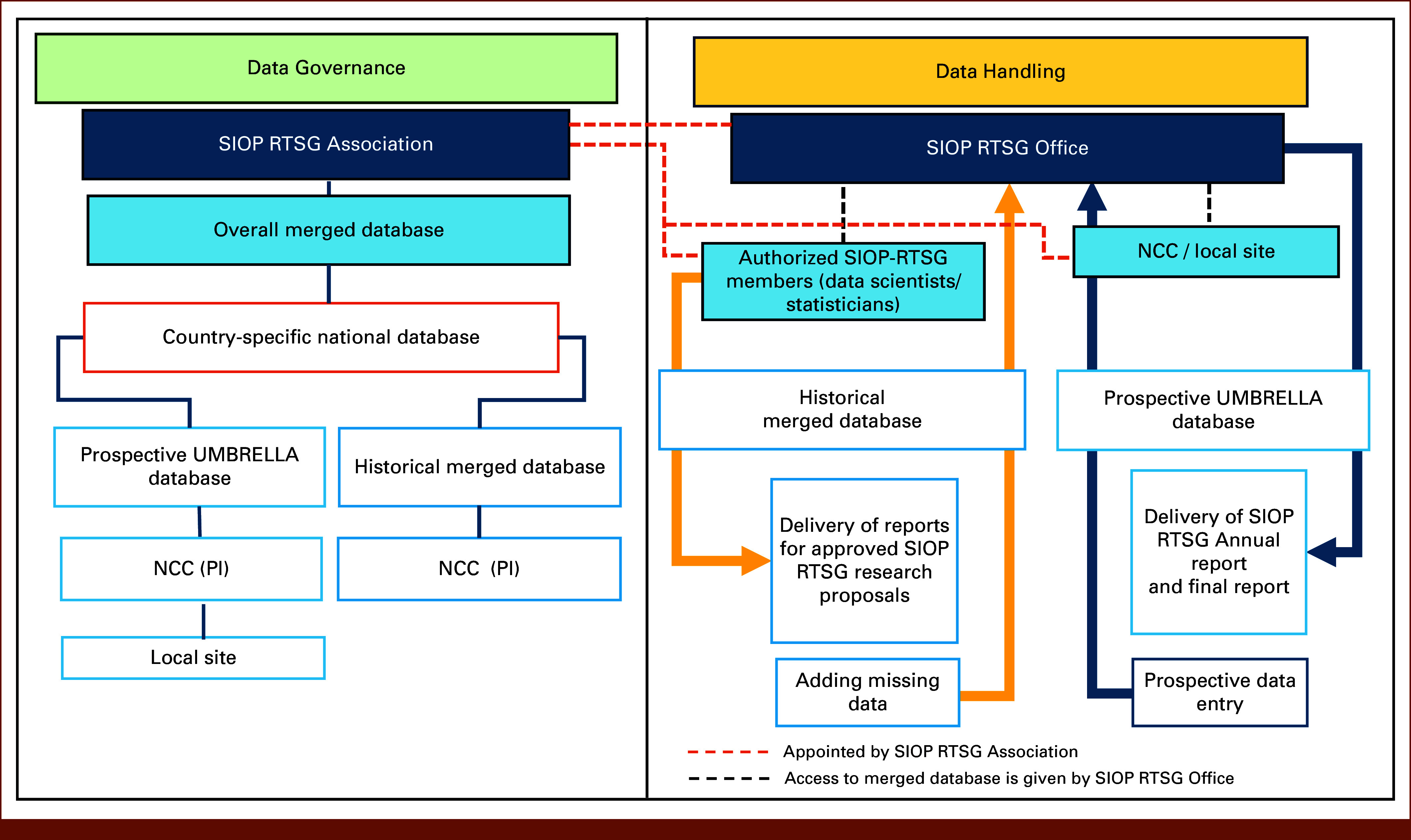
Data governance and management plans. NCC, national coordinating center; NCC (PI), Principal investigator from the coordinating center of each country; SIOP-RTSG, International Society of Pediatric Oncology Renal Tumors Study Group.

The SIOP-RTSG association is the owner of the data in this merged database. All the countries which participated in the historical studies can access their national patient data through a national coordinating center, as in the UMBRELLA study. Written agreements between the SIOP-RTSG association and national coordinating centers will govern this process (Fig 3). Research proposals can be submitted to SIOP-RTSG; upon approval by an established process, the data handling and analysis will be accomplished for these studies in a given time frame (Fig 3).

## DISCUSSION

Tumors in children are rare, and renal tumors account for only 6% of patient cases.^6^ For this reason, it is important to collect as much data as possible in a standardized way through international cooperation. Only then can meaningful information about diagnosis, treatment, and outcomes be obtained. Bringing together prospectively collected comprehensive data on diagnosis, treatment, and outcome is extremely important because survival of these children is generally good with treatments that have been available for a long time. Therefore, most of the gains will come from better characterization of the risk groups, which will allow treatment to be reduced in some groups or intensified in a few patient cases. This is only achieved with extensively collected data on large patient samples.

The SIOP-RTSG has long been aware of this and has been conducting collaborative international studies for more than 50 years to improve outcome and optimize treatment.^2^ Systematic data collection began in the early 1990s.^1^ Until recently, these data were stored in disparate systems and the analysis was complex, error prone, and time consuming. Hence, these data on more than 10,000 renal tumors is currently being brought together in a cloud-based application to preserve and make it easy to access. This will also secure the data for future research according to the FAIR (Findability, Accessibility, Interoperability, and Reusability) data principles^7,8^ for the SIOP-RTSG association and beyond. It ensures more efficient identification of missing data or inconsistencies, facilitating the improvement of data quality.

The database also provides a solid basis for collaborative or comparative studies, for example, with the Children's Oncology Group Renal Tumor Group in the transatlantic HARMONICA collaboration.^9^ Initiatives to compile retrospective data into a common format for pediatric tumors are not new.^10-12^ However, for the SIOP-RTSG, this was an opportunity to compile the data according to the format used in the ongoing prospective clinical trial (currently registered more than 4,000 patients), allowing for optimal internal data linkage. In future, the dataset will be enriched with specifically relevant biologic data, as is the primary goal of the UMBRELLA trial. Eventually, if desired, we could in one step convert the historical and current studies into a common international data standard,^13^ making international collaboration feasible.

Collecting data over a period of more than 30 years and analyzing them also has its limitations. As knowledge has increased, certain topics have been studied in more detail over time, For example, the collection of data on bilateral WT. The original systems did not allow for more records per patient (eg, surgery, pathology, and follow-up). Later, however, these capabilities were added, and more sophisticated analyses could be performed. In addition, the translation of each and every item over time was not always unambiguous. The mapping of old to new items was done in collaboration with the various disciplines to the best of our ability. In addition, it is not always possible to retrieve all missing values or to resolve inconsistencies for closed studies. Data from patients from more than 30 years ago can no longer be retrieved or are difficult to retrieve. This is partly a result of changes in hospital archives, official regulations, or privacy reasons. In the 1990s, the ethical and privacy rules were different, and different countries still have different rules in this regard. According to the GDPR,^3^ patient's initials and date of birth are considered sensitive personal data and had to be pseudonymized in the database. A disadvantage of this is that communication about patients has to go through the data protection gateway, making it more complex to identify and, in the long term, complete missing data. Finally, an important aspect of long-term data collection is more medical in nature. Whereas previously ultrasound was the most common method of imaging, currently computed tomography and magnetic resonance imaging are recommended, which may have led to a stage shift. As abnormalities can be detected more accurately, for example, in the lungs, there may have been a shift from localized disease to stage IV disease in newer patients. However, the strength includes a sustainable archival of a rich, prospectively collected, and ethically consented dataset, with similar treatment background, over three decades of consecutive historical SIOP-RTSG studies, ensured by pediatric renal tumor experts.

In summary, the merged SIOP-RTSG database in a cloud application, containing data of more than 10,000 patients with renal tumor of the past 30 years with information on diagnosis, treatment and outcome, will ensure long-term availability in a secure, flexible, and responsible manner, in line with the FAIR principles. This will make data analysis more efficient, and the data can be easily combined with those from prospective studies. This will greatly contribute to a better understanding of the characteristics and outcomes of children with renal tumors, including rare subtypes.

## Data Availability

A data sharing statement provided by the authors is available with this article at DOI https://doi.org/10.1200/CCI-25-00390.

## APPENDIX

**TABLE A1.** Description of Changes of Diagnostic Definitions, Classification, and Treatment Developments on the Basis of Increasing Scientific Knowledge (Including Variations per Countries), with Impact on Registration in Consecutive SIOP-RTSG Studies and Trials Since 1993

Time Period	1993 to 2001	2001 to 2018	2012 to 2018	2019 to Ongoing
Study Names	SIOP-93-01^a^	SIOP 2001^a^^,^^b^	UK IMPORT Study^a^^,^^b^	SIOP-RTSG-2016 UMBRELLA^a^
Diagnostic recommendations				
Pathology	No central rapid pathology review (CPR) (in GPOH since 1993)	CPR review advised, but not mandatory (in GPOH since 1993, in NL since 2001, the United Kingdom offered since 2002)	CPR advised but not mandatory (take-up rate almost 100%)	Rapid pathology and radiology review is standard of care or strongly advised
Radiology	Standard of care: US (and CT/MRI abdomen and central review in GPOH) and CXR	Standard of care: US and abdominal CT (in GPOH and Poland abdominal CT or MRI was done) and CXR (CT chest if possible in GPOH)	CRR universally offered (take-up rate almost 85% for diagnostic scans, and 70% for preop time point scans)	Standard of care: Advised MRI (and US) abdomen, while CT abdomen is acceptable. CT chest is strongly recommended and In bilateral disease MRI strongly advised. Central radiology review is mandatory for all patients before diagnosis and after preoperative chemotherapy
Stratification definitions				
Histology	BT not used as HR stratifying factor but regarded IR	BT used as HR stratifying factor	As SIOP WT 2001	Same as SIOP 2001
	Completely necrotic WT moved to low-risk tumors. FA and DA regarded as high risk	FA regarded as intermediate risk. DA remained high risk	As SIOP WT 2001	Same as SIOP 2001
Protocol patients	Nonprotocol patients: children <6 months adults >18 years non-WT relapsed disease surgical emergency/direct surgery stage V nephroblastomatosis	Nonprotocol patients: As in SIOP-93-01	All patients with renal tumors of all age groups were eligible for registration. Children younger than 6 months recommended to be treated with up-front surgery	All patients with renal tumor included Children younger than 6 months treated with up-front surgery, further refinement in 2022, and adults included in the registry
Stage definitions	Stage II with LN+ was called stage II LN + but treated as stage III	Stage II with LN+ was called stage III	As SIOP WT 2001	Same as SIOP 2001
	Stage II definition included infiltration in/beyond the tumor capsule, hilar nodes, periaortic nodes, vessels outside kidney and ureter	Stage II definition: Viable tumor infiltration in the sinus or in the perirenal fat only regarded as positive	As SIOP WT 2001	Stage II definition: Same as SIOP 2001
	Stage IV, lung metastases based on CXR	Stage IV, lung metastases based on CXR; CT scan is used only in case of doubt, in case the CXR is negative but CT positive, they are considered as pulmonary micrometasases (CT only)	For stage IV: CT chest mandated for imaging chest, CT or MRI abdomen. Local clinician discretion for defining presence of metastatic disease, no number of nodule size specified	New classification of lung lesions by chest CT Scan: All lesions <3 mm—Localised disease “CT only” Lesion(s) 3-5 mm—Metastatic disease Lesion(s) >5 mm—Definite metastatic disease
	LN positive includes both viable and nonviable tumor. LN positive is regarded as stage III	As SIOP-93-01	The presence of necrotic tumor or chemotherapy-induced changes in a lymph node or at the resection margins is regarded as proof of previous tumor with microscopic residue and therefore the tumor is assigned stage III	LN positive includes viable and nonviable tumor Nonviable tissues also called necrotic at the resection margins is NOT regarded as stage III
Treatment (Components)				
Random assignments	Stage I IR: postoperative AV1 versus AV2 (18 weeks *v* 4 weeks)	Stage I HR, stage II and III IR: AVD versus AV 27 weeks postoperative	No random assignments	No random assignments
Single-arm treatment recommendations	Stage II and III IR: postoperative 27 weeks AVD	Stage II/III IR: AV2 After closure RCT postoperatively AV2 (excluding doxorubicin was recommended (as of 2012))	Risk stratification for both preop and postop chemotherapy according to national CCLG clinical guidelines (based on SIOP WT2001 standard arms)	Stage II and III IR: AV 27 weeks postoperative VA, doxorubicin omitted (tumor volume at surgery <500 mL, nonstromal, nonepithelial)
Other treatment recommendations	Stage IV, local stage III, totally necrotic WT: no clear RT guideline	Stage IV, local stage III, totally necrotic WT: no clear RT guideline		After 2017: in case of stage IV totally necrotic WT and complete metastatic response, abdominal RT omitted in stage IV and local stage III
	Suggestions to manage relapsed WT was provided	Few treatment recommendations for relapsed WT (UK had UKWR trial open until 2005 and continued standard risk stratified treatment arms) GPOH had three treatment groups according to risk profile	National relapse treatment based on relapse national clinical trial named as UKWR	Updated treatment recommendations for relapsed WT, based on previously administered treatment
	No treatment guidelines for non-WT	Treatment guidelines for certain non-WT: CMN, CPDN, CCSK, MRTK		Treatment guidelines for all non-WT
	Children younger than 6 months and adults and non-WT regarded as nonprotocol patients	Infants and adults were regarded as nonprotocol patients	Children younger than 6 months were registered in the protocol	Children younger than 6 months and adults were regarded as protocol patients
	There was no definition for CT only patients	CT only metastasis patients were supposed to receive preoperative chemotherapy as localized disease^b^	Definition of metastasis left to clinician's discretion (national guidelines emphasised that patients with CT only lung metastasis had worse outcome in SIOP 2001 analysis)	CT only patients treated as stage IV if the definition of lung metastases was met
Stage V	Regarded as nonprotocol patient: Postoperatively treatment is done as per highest local stage. In case stage III RT recommended	Postoperative treatment as at least IR stage II with 4 weeks (this should be 27 weeks, not 4) of AV; intensification to AVD in case of unsatisfactory response	Treat at least as IR stage II. 1 year maintenance in case of radiological nephrogenic rests; intensification to CE if no response	Treat at least as IR stage II and 1 year maintenance in case of histologic NR 6 weeks of AV; intensification to CE
Nephroblastomatosis	Only definition and follow-up criteria are mentioned	1 year AV every 4 weeks if CR/3 weeks if PR, after induction phase		Switch to 6 weeks cycles induction 1 year AV every 4 weeks if CR/3 weeks if PR after induction phase
Surgery guidelines	General surgical recommendations	Surgical recommendations including NSS, stage IV and V	Same as SIOP WT 2001	Detailed surgical recommendations as per tumor types and stages, also for MIS and NSS, metastatic disease and relapses
RT	General conventional RT recommendations per site for each risk group.	Conventional RT guidelines as in 93-01	Conventional RT guidelines only (UK threshold to omit whole lung RT different recommendation to only omit if CR to preop chemo)	Detailed RT recommendations as per tumor type/stage IMRT guidelines added in 2021 Adjusted doses for HR, CCSK (lowered), whole abdomen and lungs
Boost RT	Stage II LN + or stage III: flank irradiation with boost (10-15 Gy) HR 5-10 Gy in macroscopic residual disease Lung 5-10 Gy boost	LN+: flank irradiation + boost (boost was omitted in 2013)	Stopped recommending boost since 2013 same as SIOP WT 2001	No boost in case of LN+
Differences between countries	GPOH cohort: Use of doxorubicin. All other countries: Use of epirubicin	All countries use doxorubicin (randomly assigned question)	Not applicable	All countries use doxorubicin
	Tumor volume was not used as stratification factor	GPOH: Use of preoperative tumor volume (>500 mL) as stratification factor for HR for IR (nonstromal, nonepithelial)^b^	Not applicable	All countries use of preoperative tumor volume >500 mL to augment risk stratification for IR stage II and III HR stratification (nonstromal, nonepithelial; 2, 4)
	Non-WT not routinely registered	Non-WT was recommended to register until surgery. In GPOH the outcome is also recorded for this group	Non-WTs registered but data collection is not well specified	Non-WT registered and all details of management and outcome are captured
		For most countries registration continued at a lower rate in the database even after the randomly assigned question was answered for 2 years. During this period of only registration certain countries registered non-WTs	Not applicable	All patients all non-WTs are registered with exception of MRTK which is partly registered in EU-RHAB and EpSSG
	For nephroblastomatosis GPOH had a recommendation for 2 years every 6 weeks AV and induction phase pretty like the SIOP 2001	As above	As above	As above
	Standard of care: No biopsy	UK joined SIOP-RTSG for the first time and performed percutaneous needle biopsies routinely until 2017. After publication 2017, in all countries only patients meeting clinical criteria requiring biopsy had this performed	Not applicable	Biopsies under specific conditions, refined biopsy strategy since 2022
	Radiotherapy	UK had RT in all case of stage IV with no CR after 6 weeks preop chemotherapy	Not applicable	RT in all IR patient cases with no CR after 10 week of postop chemotherapy
		RT was given for all patient cases with HR primary, but for BT not obligatory (in case of excellent response to chemo), therefore variably used in BT	Not applicable	HR patient cases always RT

Abbreviations: AV1, 4 weeks of postoperative vincristine and actinomycin D; AV2, 27 weeks of postoperative vincristine and actinomycin D; BT, blastemal type; CCSK, clear cell sarcoma of the kidney; CMN, mesoblastic nephroma; CPDN, cystic partially differentiated nephroblastoma; CR, complete response; CT, computed tomography; CXR, chest X-ray; DA, diffuse anaplasia; FA, focal anaplasia; GPOH, German Society of Pediatric Oncology and Hematology; HR, high-risk histology type of Wilms tumor; IMRT, intensity-modulated radiation therapy; IR, intermediate-risk histology type of Wilms tumor; IR, intermediate risk; LN, lymph node; MRTK, malignant rhabdoid tumor of the kidney; non-WT, renal tumors other that Wilms tumor; NR, nephrogenic rest; NSS, nephron-sparing surgery; PA, anterioposterior-posteroanterior applied conventional radiotherapy; PR, partial response; RCT, randomized controlled trial; RT, radiotherapy; WT, Wilms tumor.

a Version of protocol referred (SIOP-9301 = Protocol version from 1995, SIOP 2001 = Protocol version from 2005, UK-IMPORT = Version 4 from April 2014, UMBRELLA = version 3.2 from May 2022).

b SIOP 2001 included the United Kingdom as a study center between 2005 and 2010; later the United Kingdom had started registering in the UK IMPORT study while the other countries continued registration in SIOP 2001 until 2019.
